# Intranasal VACV VR-1354 infection impairs chemosensory function and induces olfactory bulb neuroinflammation in mice

**DOI:** 10.3389/fimmu.2026.1759456

**Published:** 2026-04-20

**Authors:** Shun Li, Yunxiang Wu, Chao Wang, Hanqing Wu, Lin Yang, Lixiang Chen, Hua Yang, Jun Zhang, Xiaohui Zhou

**Affiliations:** 1 Department of Animal Model, Shanghai Public Health Clinical Center, Fudan University, Shanghai, China; 2 Shanghai Institute of Infectious Diseases and Biosecurity, Fudan University, Shanghai, China; 3 School of Life Sciences, Shanghai Normal University, Shanghai, China

**Keywords:** Blood-brain barrier, intranasal infection, neuroinflammation, olfactory dysfunction, VACV VR-1354

## Abstract

Vaccinia virus (VACV) and monkeypox virus (MPXV) are closely related members of the family Poxviridae, genus Orthopoxvirus, both capable of causing systemic infections with potential neurological complications. Although live, replication-competent VACV strains were historically used in smallpox vaccination, their clinical use was associated with rare but severe central nervous system (CNS)-related adverse events. Despite this, the mechanisms underlying VACV-induced CNS pathology, particularly olfactory dysfunction, remain poorly characterized. In this study, we found VACV-VR1354, a tissue culture-adapted derivative of the neurovirulent Western Reserve strain, can invade the CNS via the olfactory route and induce olfactory impairment. By using an intranasal infection model in two inbred mouse strains—C57BL/6N and BALB/c, we demonstrate that VACV-VR1354 efficiently disseminates from the nasal mucosa to the brain, as evidenced by a spatiotemporal gradient of viral DNA load (nasal mucosa > olfactory bulb > cerebrum > cerebellum). Evans blue extravasation assays indicated a transient increase in blood–brain barrier (BBB) permeability in the olfactory bulb, peaking at 7 days post-infection (dpi) and resolving by 14 dpi, with more pronounced effects in C57BL/6N mice. Neuroinvasion was accompanied by robust microglial and astrocytic activation, as well as injury to mature olfactory sensory neurons, particularly at 7 dpi. Transcriptomic profiling of the olfactory bulb revealed significant downregulation of olfactory receptor (OR) genes, with the downregulated genes significantly enriched in olfactory transduction pathways. Concurrently, strong upregulation of proinflammatory cytokines, chemokines, and interferon-stimulated genes (ISGs) was detected in the olfactory bulb tissue, indicative of intense neuroinflammation. Behaviorally, infected C57BL/6N mice exhibited impaired aversion to camphor odor between 14 and 49 dpi, with full functional recovery observed by 56 dpi. Collectively, our findings showed that intranasal infection of mice with VACV-VR1354 leads to a transient increase BBB permeability, neuroinflammation, and reversible olfactory/chemosensory impairment. This murine model recapitulates key features of post-viral olfactory loss and establishes a valuable platform for mechanistic studies of orthopoxvirus neuropathogenesis and therapeutic evaluation of interventions targeting viral neuroinvasion and sensory recovery.

## 1 Introduction

Similar to monkeypox virus (MPXV), vaccinia virus (VACV) belongs to the family *Orthopoxviridae* and possesses a linear, double-stranded DNA genome of approximately 200 kb (1, 2). Its genome contains a highly conserved central region encoding essential components of the viral replication machinery (3). Historically, licensed smallpox vaccines have been developed using live, replication-competent VACV strains propagated in calf lymph (4–7). While these vaccines were instrumental in eradicating smallpox, their use was associated with rare but serious adverse events, including central nervous system (CNS) complications such as encephalitis and postvaccinal encephalopathy (8–11). These clinical observations underscore the need to elucidate the neurotropic potential and neuropathogenic mechanisms of VACV infection.

Several mouse models—including C57BL/6J, BALB/c, CAST/EiJ, and immunodeficient strains—have been widely used to study VACV pathogenesis and evaluate antiviral agents and vaccine candidates (12–15). Among VACV strains, the laboratory-adapted Western Reserve (VACV-WR) strain is particularly notable for its neurovirulence and neuroinvasiveness (16, 17). Notably, Aude et al. (18) demonstrated that following intravenous inoculation, VACV-WR can cross the blood–brain barrier (BBB) in C57BL/6 mice; however, overt encephalitis manifests primarily in the presence of bacterial co-infection. Despite these findings, the mechanisms underlying VACV-induced CNS pathology—particularly neuroinflammation, neuronal damage, and functional neurological deficits—remain incompletely understood.

Recent studies have revealed that MPXV potentially invade the CNS via the olfactory epithelium. Following intranasal exposure, MPXV rapidly replicates in the nasal septum and respiratory mucosa before spreading to the brain parenchyma, implicating the olfactory pathway as a direct conduit for neuroinvasion (19). Given the close phylogenetic relationship between MPXV and VACV within the *Orthopoxviridae* family (20, 21), we hypothesized that VACV-WR, and its tissue culture-adapted derivative VACV-VR1354, may similarly exploit the olfactory neuroepithelium to access the CNS. The olfactory system provides a unique and vulnerable route connecting the external environment directly to the brain, enabling certain neurotropic pathogens to bypass classical protective barriers such as the BBB (22–24). However, the evidence that VACV can affect the olfactory epithelium, the molecular and cellular mechanisms—including direct neuronal infection, neuroinflammatory activation, BBB disruption, and resulting olfactory dysfunction—are poorly defined. Furthermore, the neuroinvasive capacity of VACV-VR1354, its impact on olfactory bulb homeostasis, and the dynamics of host–pathogen interactions within this neural tissue have not been systematically characterized.

In this study, we established an intranasal infection model using VACV-VR1354 in both C57BL/6N and BALB/c mice to comprehensively assess the virological, histopathological, transcriptomic, and behavioral consequences of infection. We monitored viral dissemination into the CNS, BBB integrity, neuroinflammatory responses, transcriptional changes in the olfactory bulb, and olfactory function over time. Our results demonstrate that VACV-VR1354 efficiently invades the CNS via the olfactory pathway, triggering transient BBB disruption, robust neuroinflammation, downregulation of olfactory receptor genes, and reversible olfactory dysfunction. Our findings provide direct evidence that VACV can exploit the olfactory epithelium to invade the CNS and offer broader implications for understanding the mechanisms of post-viral olfactory dysfunction. Moreover, the VACV-VR1354 murine model established in this study represents a valuable tool for investigating the pathogenesis of olfactory injury and recovery, as well as for evaluating potential therapeutic interventions.

## 2 Materials and methods

### 2.1 Virus propagation and concentration

Vaccinia virus VR-1354 (NIH TC-adapted; ATCC^®^ VR-1354™) was obtained from the American Type Culture Collection (ATCC, Manassas, VA, USA) and kindly provided by the laboratory of Prof. Dongming Zhou at the Shanghai Public Health Clinical Center, Fudan University. Vero cells were seeded into T25 flasks and cultured with 10 mL of DMEM supplemented with 10% fetal bovine serum (FBS) at 37 °C for 2 days. The culture medium was discarded and replaced with 10 mL of DMEM containing 2% FBS; the virus was then inoculated at an MOI of 0.01 and adsorbed at 37 °C for 90 min. After adsorption, the medium was replaced with 10 mL of fresh DMEM containing 2% FBS, and the cultures were maintained at 37 °C for 2–3 days. Following partial removal of the supernatant, adherent cells were scraped off, transferred to a 15 mL centrifuge tube, and subjected to three freeze-thaw cycles. The lysate was centrifuged at 1,200 rpm for 15 min; the supernatant was retained and the pellet discarded. Ultracentrifugation was performed at 30,000 rpm for 80 min at 4°C. The supernatant was discarded, and the viral pellet was resuspended in 500 μL of PBS, dissolved overnight, and stored at –80 °C.

### 2.2 Plaque assay for vaccinia virus VR-1354

Vero cells were seeded into 6-well plates and incubated at 37 °C until confluent monolayers formed. Test samples were serially diluted tenfold in DMEM containing 2% FBS. The culture medium was removed from the wells, and 1,000 μL of each dilution was added per well and adsorbed at 37 °C for 90 min. After adsorption, unbound virus was removed by washing each well three times with DMEM containing 1% FBS. Subsequently, 500 μL of overlay medium (DMEM with 3% FBS, 1% penicillin-streptomycin, and 1% agarose) was added to each well. Once the agarose-containing medium had solidified, the plates were incubated at 37 °C for 60 h until distinct plaques appeared. Then, 250 μL of 0.5% crystal violet staining solution was added to each well and left to stain overnight at room temperature. The agarose overlay was removed, plaques were counted, and viral titers were calculated based on the corresponding dilution factors.

### 2.3 Mice and ethics statement

Female C57BL/6N mice (Catalog Numbers B203-01) and female BALB/c mice (Catalog Numbers B201-01), specific pathogen-free (SPF) grade, aged 4 weeks and weighing 12–14 g, were obtained from SPF (Beijing) Biotechnology Co., Ltd. [SCXK (Beijing) 2019-0010]. All mice infected experiments were implemented in Biosafety Level 2 (BSL-2) laboratory at the Shanghai Public Health Clinical Center. This study was approved by the Ethics Committee of the Shanghai Public Health Clinical Center (Approval No. 2025-A016-01). The mice housing conditions were as follows: temperature 22–25 °C, relative humidity 50–70%, 12-hour light/dark cycle, with food and water provided ad libitum. The weight loss of mice more than 25% was determined as a humane endpoint. All methods were carried out in accordance with relevant guidelines and regulations. The study is reported in accordance with ARRIVE guidelines (https://arriveguidelines.org).

### 2.4 Animal experiments

LD_50_ of Vaccinia Virus VR-1354 for both C57BL/6N mice and female BALB/c mice were determined separately. Mice were anesthetized by inhalation of isoflurane (Abbott Pharmaceuticals, Shanghai, B506) using a cotton ball saturated with pure isoflurane, which was held gently adjacent to the nares. Anesthesia depth was confirmed by loss of righting reflex and complete flaccidity of the tail base—signs typically evident within 15–20 s—and only mice meeting these criteria proceeded to intranasal inoculation. The cotton ball was then removed, and inoculation was performed immediately. Control mice received 40 μL of PBS intranasally. C57BL/6N mice were challenged with 5×10^6^, 5×10^5^, 5×10^4^, 5×10^3^, or 5×10^2^ PFU of virus, with 8 mice per dose group, respectively. BALB/c mice were challenged with 1.25×10^4^, 2.5×10^3^ or 5×10^2^ PFU of virus, with 8 mice per dose group, respectively. After infection, body weight and survival status of mice were recorded daily for 14 consecutive days. The LD_50_ values were calculated using the Karber method. The formula applied is:LD_50_=Xk-i×(∑P-(3-Pm-Pn)/4). Where: (X_k) = the logarithm (base 10) of the highest dose administered; (i) = the interval (in log_10_ units) between adjacent doses; (P) = the mortality rate (as a decimal) observed at each dose level; (P_m) = the highest mortality rate among all groups; (P_n) = the lowest mortality rate among all groups. The LD_50_ was determined to be 2.8×10^3^ PFU in BALB/C mice and 2.5×10^3^ PFU in C57BL/6N mice. Twenty-four BALB/c and twenty-four C57BL/6N mice were inoculated intranasally with 2.5×10^3^ PFU of Vaccinia virus (strain VR-1354). Corresponding control groups received 40 μL of phosphate-buffered saline (PBS) via the same route. Mice were monitored daily for body weight changes and survival over a 14-day period post-infection. Tissue samples were collected at 3, 7, and 14 days post-infection for subsequent analysis. At the end of the experiment, mice were humanely euthanized by CO_2_ inhalation. The CO_2_ flow rate was maintained at 40–50% of the chamber volume per minute, and the total exposure duration was 6.0 ± 0.5 minutes. Death was confirmed by verification of three objective endpoints: cessation of respiration for ≥1 min, absence of heartbeat (confirmed by auscultation or palpation), and loss of corneal and pupillary light reflexes.

### 2.5 Quantification of viral DNA

Total DNA was extracted from each tissues using the MolPure Viral DNA/RNA Kit (Yeasen Biotechnology, Shanghai, Cat. No. 19321ES50) according to the manufacturer’s instructions. Bioer LineGene 9600 Plus real-time fluorescent PCR system (FQD-96, Hangzhou Bioer Technology Co., Ltd., China) was used for qPCR with the following primers: 5’ GCTAAAAGCGACGTCTTGTATT 3’ (forward) and 5’ GTGTCATTTGTAGTTGATGTCA 3’ (reverse). A virus-specific qPCR standard curve was constructed, yielding the equation: viral load = 10^[(28.63−CT)/3.49], R2 = 0.9882. Viral loads for experimental samples were then calculated by substituting their respective CT values into this equation.

### 2.6 Evans blue assay

Mice were euthanized at 3, 7, and 14 days post-viral infection for assessment of blood-brain barrier (BBB) permeability. Evans blue dye (2%) was administered via tail vein injection at a volume of 4 μL per gram of body weight. After 30 minutes of systemic circulation, mice were anesthetized by intraperitoneal injection of 2.5% (w/v) tribromoethanol at 10 μL/g body weight. A thoracotomy was performed to expose the heart, and the right atrium was incised to establish an outflow tract. A cannula was inserted into the left ventricle, and transcardial perfusion was initiated with cold phosphate-buffered saline (PBS) until the effluent from the right atrium became clear, indicating complete removal of intravascular blood. This was followed by perfusion with 15 mL of 4% paraformaldehyde (PFA) in PBS for whole-body fixation. After fixation, perfusion was terminated, and the brain was carefully dissected out. The isolated brain tissues were examined under a stereomicroscope, and representative images were captured for further analysis.

### 2.7 Immunofluorescence staining

Whole mouse brains were collected at 3, 7, and 14 days post viral infection and immediately fixed in 4% paraformaldehyde solution. Fixed tissues were then processed for cryosectioning, followed by immunofluorescence staining, imaging, and analysis. The antibodies used and their respective concentrations were: Iba-1 (Abcam, AB254360, 1:1000), GFAP (Servicebio, GB15100-100, 1:2000), OMP (Wako, 544-10001, 1:800), Alexa Fluor 488 (ThermoFisher, A-11008, 1:1000), Alexa Fluor 594 (ThermoFisher, A-11012, 1:1000).

All immunofluorescence images were acquired using a confocal laser-scanning microscope, with imaging parameters optimized for each fluorophore and rigorously held constant across all experimental samples to ensure quantitative comparability and inter-group consistency. For region-of-interest (ROI) definition, GFAP- and IBA-1–stained sections were analyzed primarily within the glomerular layer of the olfactory bulb, with additional inclusion of adjacent anatomical subregions—including the external plexiform layer, mitral cell layer, internal plexiform layer, and granule cell layer—to capture broader glial activation patterns; in contrast, OMP-stained sections were analyzed exclusively within the glomerular layer to specifically assess olfactory receptor neuron terminal integrity. All tissue sections were cut at a uniform thickness of 15 μm, and one section per animal was selected for quantitative analysis, with three independent biological replicates (three animals) included per experimental group. To eliminate observer bias, a strict double-blind protocol was implemented: one investigator assigned randomized alphanumeric codes to all slides prior to imaging, and a second independent investigator—blinded to group identity—performed all image acquisition and subsequent quantitative analyses. Confocal imaging settings were systematically tailored to each antibody, fluorophore, and mouse strain combination as follows: for BALB/c mice, IBA-1 signal was captured with a pixel dwell time of 3.85 μs, gain of 70%, scan speed of 200 Hz, 499 nm laser line intensity of 6.99%, pinhole set to 2 Airy units, image resolution of 1024×1024, and 20× magnification; GFAP signal used identical dwell time, scan speed, laser line, pinhole, and resolution, but with gain increased to 85% and laser intensity reduced to 5.19% at 10× magnification; OMP signal employed a longer pixel dwell time of 7.6875 μs, gain of 85%, slower scan speed of 100 Hz, 578 nm laser line intensity of 3.48%, pinhole of 1 Airy unit, and 1× magnification—maintaining full-resolution acquisition (1024 × 1024). For C57BL/6 mice, IBA-1 and GFAP imaging matched the corresponding BALB/c settings except for gain adjustments (80% for IBA-1 and 70% for GFAP); OMP imaging retained the same pinhole (1 Airy unit), resolution (1024 × 1024), and magnification (1×), but used a pixel dwell time of 3.85 μs, gain of 80%, and scan speed of 200 Hz to accommodate strain-specific signal intensity and background characteristics.

### 2.8 Behavioral experiments

Mice infected with VACV and uninfected control mice underwent a Conditioned Place Preference (CPP) test to investigate potential olfactory deficits. The apparatus consisted of a standard three-chamber CPP system, comprising two lateral chambers and a central chamber. From a cohort of VACV-infected mice, 24 animals were randomly selected for behavioral testing. Behavioral assays began at 14 days post-infection (dpi), following confirmation of normal motor function. Subsequent tests were conducted weekly through 56 dpi. Prior to each behavioral session, mice underwent a 30-minute habituation period in the apparatus without odor cues; locomotor trajectories were recorded during this period to confirm absence of innate side preference.

Then, each mouse was gently placed in the central chamber, and access to the lateral chambers was temporarily blocked by closing opaque dividing doors. A custom-built scent box containing 0.05% (v/v) camphor solution in mineral oil was then introduced into the left lateral chamber as the conditioned odor stimulus; an identical, unscented (blank) scent box was simultaneously placed in the right lateral chamber. After a 2-minute equilibration period, the dividing doors were removed, allowing the mouse to explore all three chambers freely for 10 minutes. During this period, a video tracking system continuously recorded the animal’s movement trajectory, spatial position, and time spent in each chamber. Time spent in each chamber and locomotor parameters were analyzed to assess conditioned place preference and olfactory-driven exploratory behavior.

### 2.9 RNA sequencing and bioinformatic analysis

Olfactory bulb tissues from virus-infected and uninfected control mice were collected following CO_2_ euthanasia. The olfactory bulbs were processed immediately for RNA extraction. Total RNA was isolated using the RNeasy Mini Kit (Qiagen, Germany), quantified using a Qubit fluorometer (Thermo Fisher Scientific, Waltham, MA, USA), and assessed for integrity by agarose gel electrophoresis. High-quality RNA samples were used to construct stranded RNA-seq libraries with the TruSeq RNA Library Prep Kit v2 (Illumina, San Diego, CA, USA). Libraries were pooled and sequenced on a NovaSeq 6000 platform (Illumina) to generate 150 bp paired-end reads, with an average depth of approximately 30 million reads per sample. Raw sequencing reads were trimmed to remove adapter sequences and low-quality bases using Skewer (v0.2.2), and data quality was evaluated using FastQC (v0.12.1). The clean reads were aligned to the mouse reference genome (mm10) using STAR. The raw read counts of all annotated genes were quantified using StringTie. Differentially expression analysis between different groups were performed by the R package DESeq2 with the likelihood ratio test option. Differentially expressed genes exhibiting two-fold changes and P values < 0.05 were selected. If the gene DESeq2 normalized read count value was close to 0, log2 transformation was performed after adding 1. For differential expressed protein-coding genes, significantly enriched GO terms and KEGG pathways were separately identified by the R package TopGO. Significantly enriched GO terms and KEGG pathways were selected by a threshold p value < 0.05. The original sequencing data that support the findings of this study have been deposited into CNSA (25) with accession number CNP0008526.

### 2.10 Statistical analysis

Fluorescence intensity quantification was performed using ImageJ software (version 1.54). Original immunofluorescence images were imported into ImageJ and converted to grayscale images. The entire image was selected as the region of interest, and consistent parameters were applied across all images within the same experimental group to calculate the mean gray value, which was used to represent fluorescence intensity. For cell counting, the number of nuclei within the grayscale image was manually counted. Synaptic quantity was determined by manual counting of synapses in original immunofluorescence images. All experiments included three independent biological replicates.

Data visualization and statistical evaluation were conducted using GraphPad Prism version 10.0. Results are presented as mean values ± standard error of the mean (SEM). For comparisons between two groups, a two-tailed Student’s t-test was employed. When comparing among multiple groups, a one-way ANOVA with Tukey’s *post hoc* test was utilized to determine significance. Animal survival curves were generated using the Kaplan-Meier method, and group comparisons were conducted with the log-rank (Mantel-Cox) test. Statistical significance is indicated as follows: ns (not significant), *p < 0.05, **p < 0.01, ***p < 0.001, ****p < 0.0001.

## 3 Results

### 3.1 Construction of VACV VR-1354 infection models in C57BL/6N and BALB/c mice

**Figure 1:**
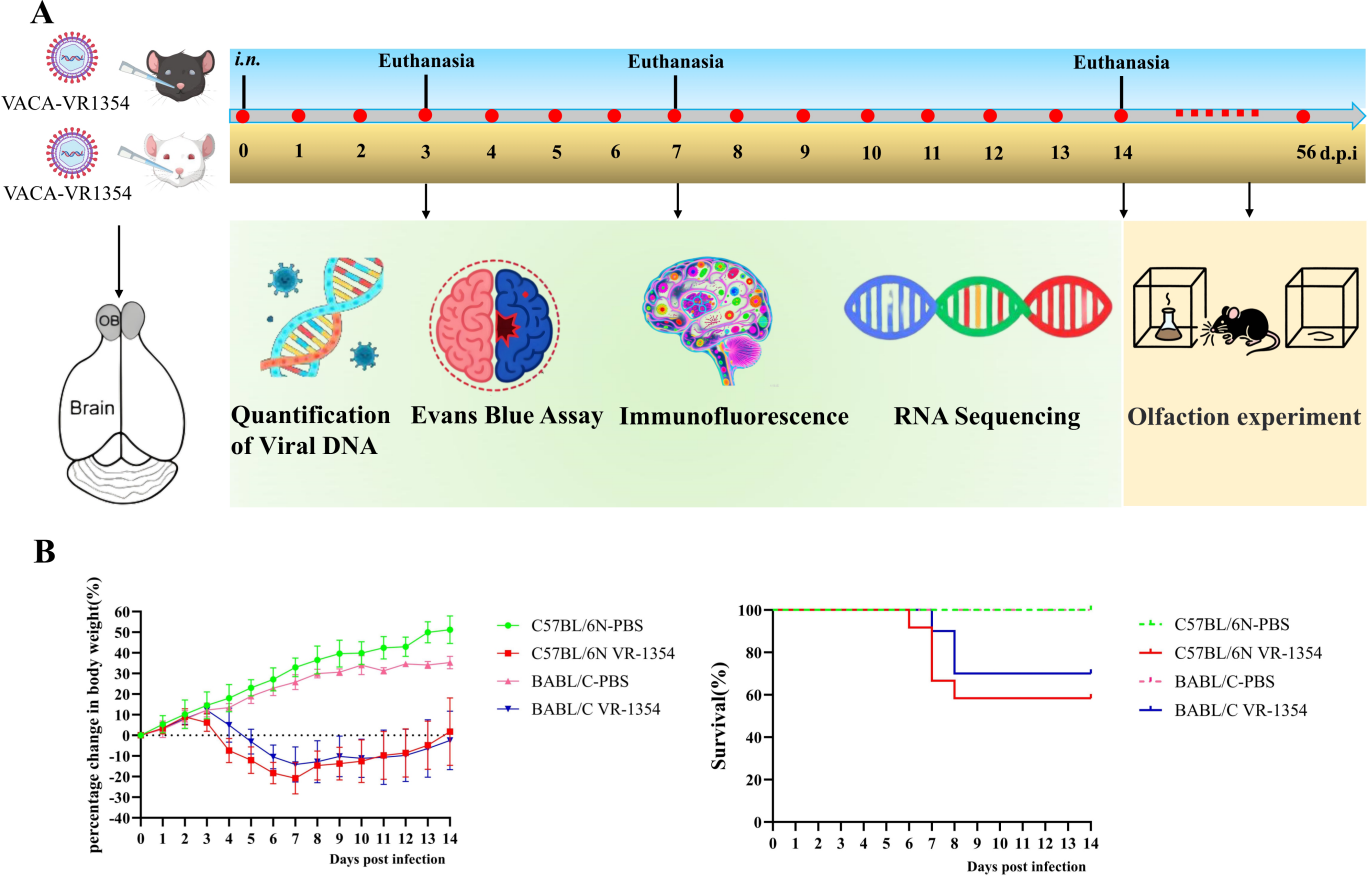
Experimental design and phenotypic assessment of intranasal VACV VR-1354 infection in mice. **(A)** Schematic timeline of the experimental procedures. C57BL/6N and BALB/c mice were intranasally (*i.n.*) inoculated with 2.5×10³ PFU of VACV VR-1354. Key endpoints are indicated: body weight and survival were monitored for 14 days post-infection (dpi); olfactory function was assessed longitudinally over 56 dpi (blue arrow); and animals were euthanized at 3, 7, and 14 dpi (red arrows) for tissue collection and subsequent analyses, including Evans Blue assay for vascular permeability, quantification of viral DNA by qPCR, bulk RNA sequencing of olfactory bulb tissue, and immunofluorescence staining. **(B)** Phenotypic outcomes of infection. (Left panel) Mean percentage change in body weight (± SEM) was calculated daily relative to pre-infection baseline (Day 0) for both mouse strains (n = 8 mice per group). (Right panel) Survival curves for C57BL/6N (χ²=6.069, *df* = 1, *p* = 0.0138) and BALB/c (χ²=3.344, *df* = 1, *p* = 0.0675) mice were plotted (n = 8 mice per group).

As in our previous study, we first determined the LD_50_ of VACV VR-1354 in C57BL/6N and BALB/c mice. The results indicated that the LD_50_ was 2.5×10^3^ PFU in C57BL/6N mice and 2.8×10^3^ PFU in BALB/c mice. Subsequently, we intranasally inoculated both C57BL/6N and BALB/c mice with an identical dose (2.5×10^3^ PFU) of VACV VR-1354, and monitored body weight, survival rate and disease signs (**Figure 1A**). As shown in **Figure 1B**, both C57BL/6N and BALB/c mice infected with 2.5×10^3^ PFU of VACV VR-1354 exhibited body weight loss, with C57BL/6N mice showing significantly greater weight reduction. Compared to the control group, infection with 2.5×10^3^ PFU of VACV VR-1354 resulted in 5 out of 12 infected animals died, yielding a mortality rate of 41.7% (5/12) in C57BL/6N mice and 3 out of 10 infected animals died, yielding a mortality rate of 30.0% BALB/c mice. Based on the body weight loss curves and survival kinetics, VACV VR-1354 exhibits higher lethality and pathogenicity in C57BL/6N mice than in BALB/c mice.

### 3.2 Evans blue assay and viral DNA loads in C57BL/6N and BALB/c mice after VACV VR-1354 infection

**Figure 2:**
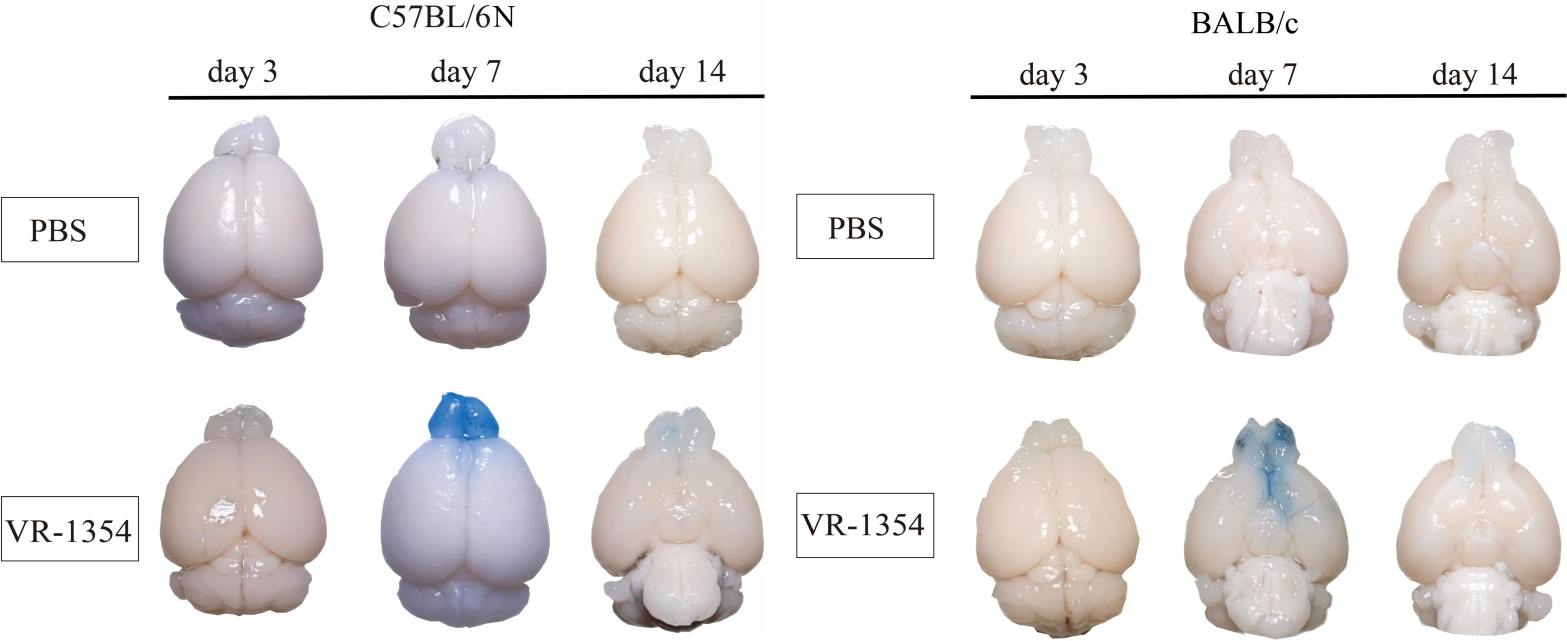
Evans blue dye leakage assay reveals temporal increase in blood-brain barrier (BBB) permeability in C57BL/6N and BALB/c mice following intranasal VACV VR-1354 infection. Experimental design timeline: C57BL/6N and BALB/c mice were infected with VACV VR-1354 or mock-infected with phosphate-buffered saline (PBS). Evans blue dye (2%, 4 mL/kg body weight) was administered via tail vein injection 0.5 hours prior to brain collection at 3, 7, and 14 days post-infection (dpi).Representative brain images: Gross morphological examination of brains harvested at 3, 7, and 14 dpi. (n = 3 mice per group).

Compared with the uninfected control group, in C57BL/6N mice infected with VACV VR-1354, blue penetration was observed in the olfactory bulb of the brain tissue. In the olfactory bulb of the brain tissue 3 days after infection, almost no blue penetration was observed. The deepest blue penetration was observed in the olfactory bulb of the brain tissue 7 days after infection, while the blue color in the olfactory bulb of the brain tissue 14 days after infection faded (**Figure 2**). Similarly, after BALB/c mice were infected with VACV VR-1354, Evans blue penetration was also observed in the olfactory bulb of the brain tissue, and the trend was basically consistent with that in C57BL/6N mice. The most severe blue penetration was observed in the olfactory bulb of the brain tissue on the 7th day after infection, almost no penetration was observed on the 3rd day after infection, and the blue penetration faded on the 14th day after infection. From the results of Evans blue staining on the 7th day after infection, it can be seen that after VACV VR-1354 infection, the degree of blue penetration in the olfactory bulb of C57BL/6N mice was more severe than that in BALB/c mice.

**Figure 3:**
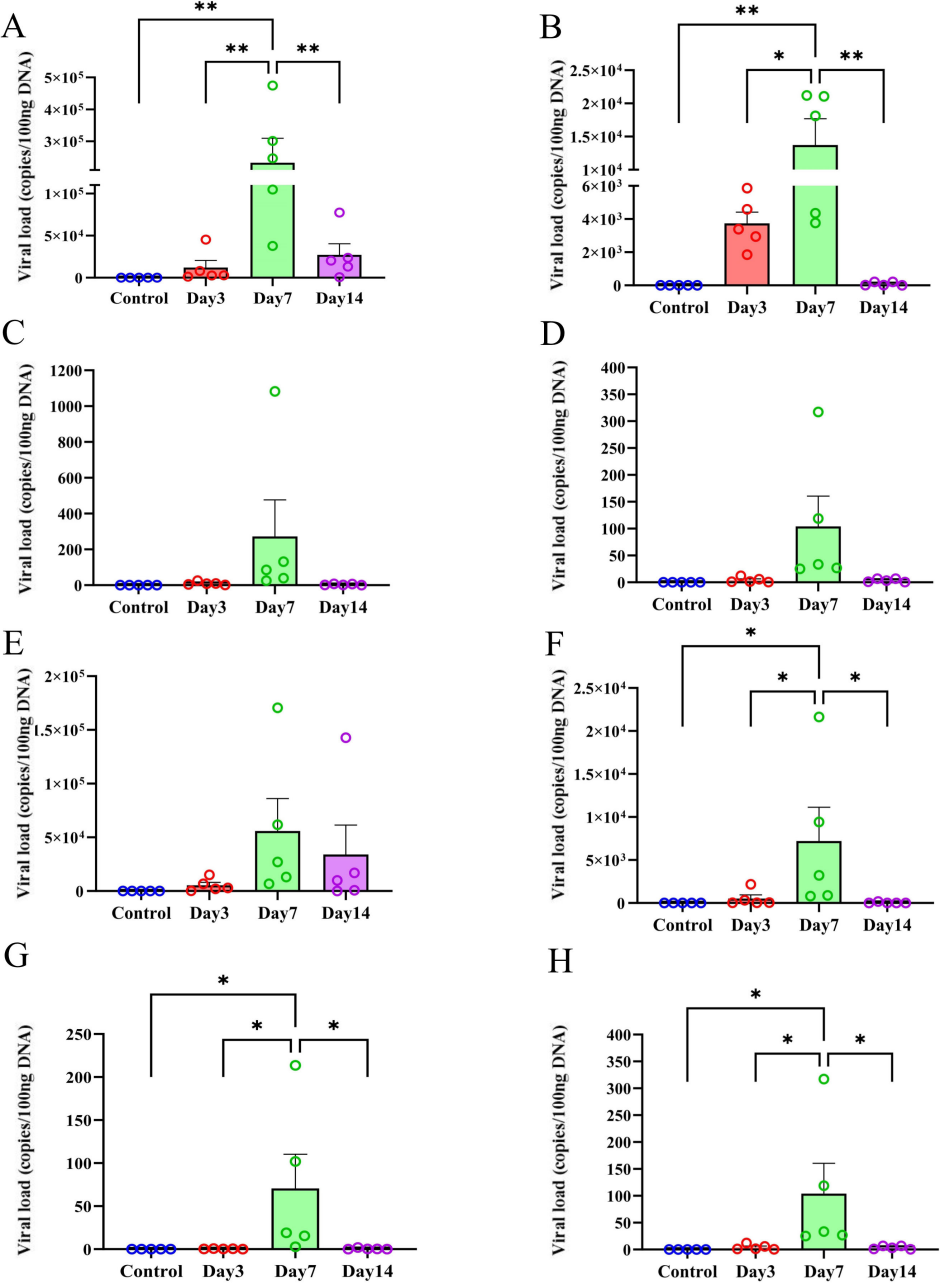
Viral DNA loads distribution in C57BL/6N and BALB/c mice after VACV VR-1354 infection. **(A–D)** Viral titers in nasal mucosa **(A)**, olfactory bulb **(B)**, cerebrum **(C)** and cerebellum **(D)** of C57BL/6N mice at 3, 7, and 14 dpi. **(E–H)** Corresponding viral loads in BALB/c mice: nasal mucosa **(E)**, olfactory bulb **(F)**, cerebrum **(G)**, and cerebellum **(H)**. (n = 5 mice per group). (**P < 0.01,*P < 0.05).

Further, the viral loads in the nasal mucosa, olfactory bulb, cerebrum, and cerebellum tissues were detected to explore the spatial distribution characteristics of the virus. After C57BL/6N mice were infected with the virus, the virus could be detected in the nasal mucosa on the 3rd day after infection. A relatively high viral load was detected in the nasal mucosa on the 7th day after infection. On the 14th day after infection, the viral load in the nasal mucosa decreased, but a large amount of viral load could still be detected (**Figure 3A**). In the olfactory bulb tissue, the virus could be detected on the 3rd day after infection. A relatively high viral load was detected in the olfactory bulb tissue on the 7th day after infection, and almost no viral load could be detected in the olfactory bulb tissue on the 14th day after infection (**Figure 3B**). In the cerebrum and cerebellum, a small amount of viral load could be detected on the 7th day after infection, and almost no viral load could be detected on the 3rd and 14th days after infection (**Figures 3C, D**). After BALB/c mice were infected with VACV VR-1354, the spatial distribution trend of viral loads in the nasal mucosa, olfactory bulb, cerebrum, and cerebellum tissues was similar to that in C57BL/6N mice (**Figures 3E–H**). However, when comparing the two strains of mice, on the 7th day after infection, the viral loads detected in the nasal mucosa and olfactory bulb of C57BL/6N mice were higher than those detected in the nasal mucosa and olfactory bulb of BALB/c mice.

These results suggest that intranasal infection of C57BL/6N and BALB/c mice with VACV VR-1354 can cause the disruption of the blood-brain barrier (BBB), mainly occurring in the olfactory bulb of the mice. The degree of blood-brain barrier disruption in the olfactory bulb of C57BL/6N mice is higher than that in BALB/c mice. The results of the spatial distribution characteristics of viral loads suggest that the spatial distribution characteristics of VACV VR-1354 infection in C57BL/6N and BALB/c mice are basically similar, but the viral loads in the same tissues at the same time points are higher in C57BL/6N mice than in BALB/c mice. In addition, in both strains of mice, the distribution of viral loads shows a gradient characteristic, that is, the viral load is the highest in the nasal mucosa, followed by the olfactory bulb, and substantially lower in the cerebrum and cerebellum.

### 3.3 Analysis of microglia, astrocytes, and nerves in the olfactory bulb of C57BL/6N and BALB/c mice

**Figure 4:**
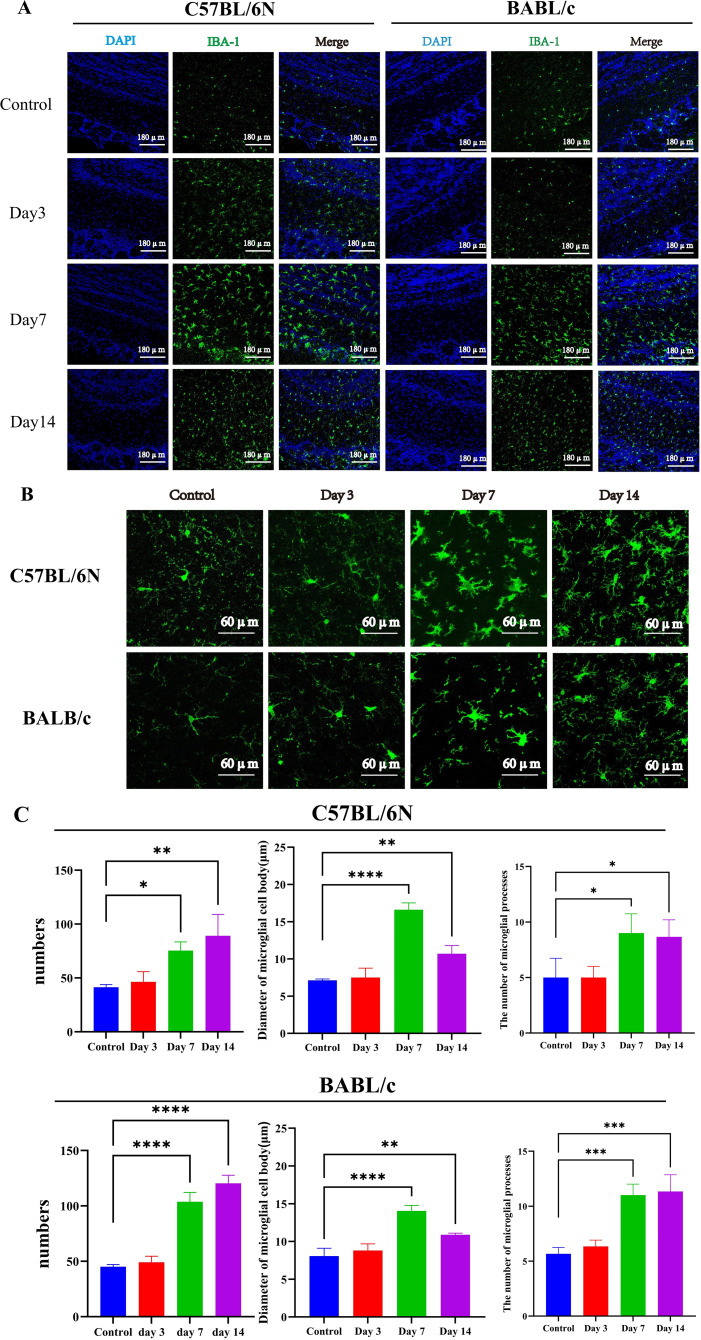
Activation of microglia in the olfactory bulb of C57BL/6N and BALB/c mice following VACV VR-1354 infection. **(A)** Immunofluorescence staining for Iba1 revealed progressive microglial activation in the olfactory bulb, characterized by increased cell density at 7 dpi and sustained elevation at 14 dpi in both strains (20× magnification). **(B)** High-magnification (63×) images displayed morphological hallmarks of microglial activation at 7 dpi, including enlarged cell bodies and retracted, thickened processes. **(C)** Quantification of the microglial activation index across time points by ImagJ. (n =3 mice per group). (****P < 0.0001, ***P < 0.001, **P < 0.01,*P < 0.05).

The microglia, astrocytes, and olfactory nerves in the olfactory bulb of C57BL/6N and BALB/c mice were examined at 3, 7, and 14 days post-infection with VACV VR-1354. In both mouse strains, microglia exhibited mild activation at 3 days post-infection (dpi), with a significant increase in cell numbers observed at 7 dpi, which remained elevated at 14 dpi (**Figure 4A**). High-magnification (63×) imaging further revealed that, at 7 dpi, activated microglia not only increased numerically but also displayed morphological hallmarks of activation, including enlarged cell bodies, increased process complexity, and shortened processes (**Figure 4B**). By 14 dpi, the degree of activation, as indicated by cell body size and process density, had begun to subside (**Figure 4C**).

**Figure 5:**
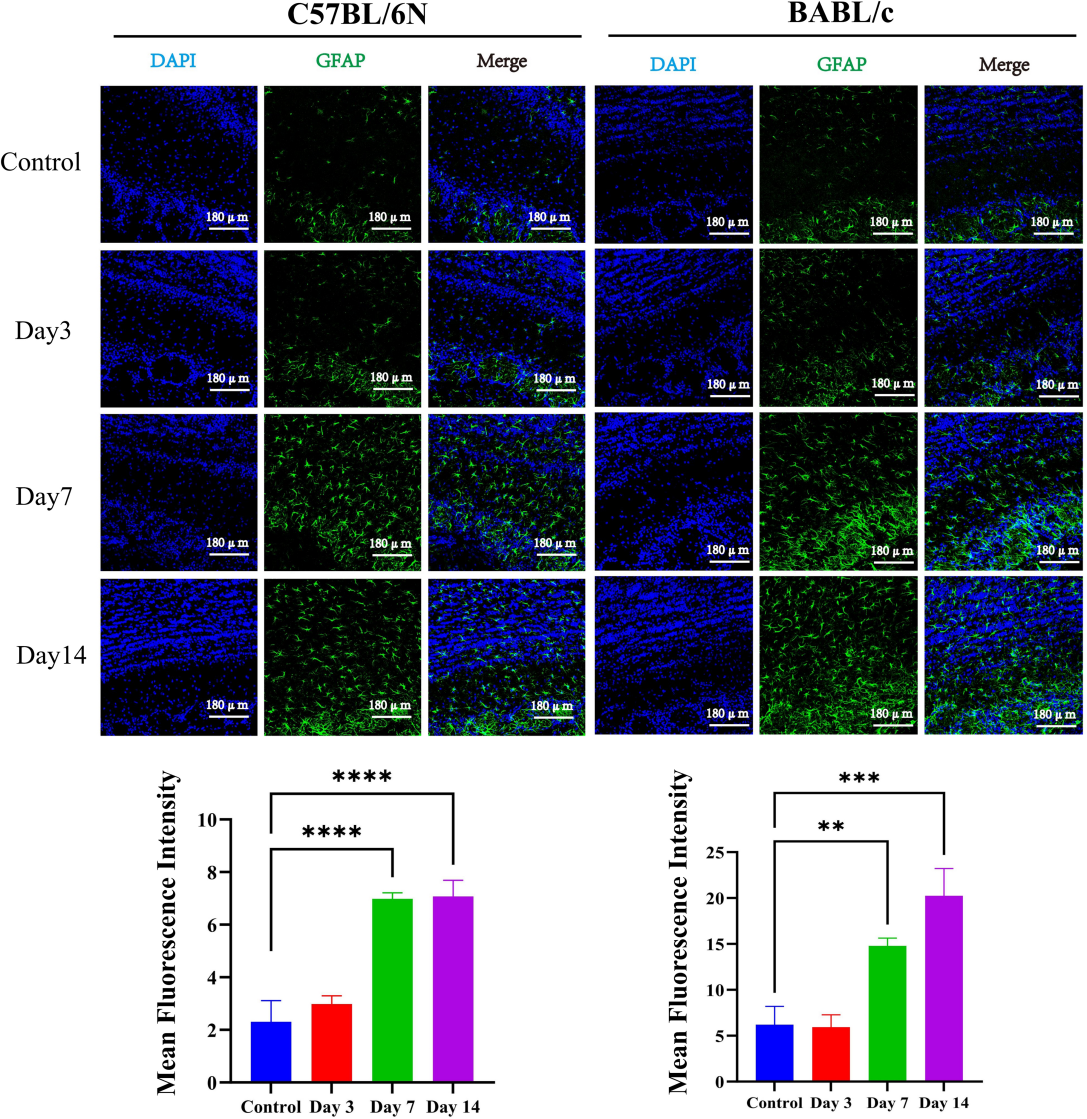
Temporal dynamics of astrocyte activation in the olfactory bulb of C57BL/6N and BALB/c mice following VACV VR-1354 infection assessed by GFAP immunofluorescence staining. (Up panel) Representative immunofluorescence images of olfactory bulb sections stained with DAPI (blue, nuclear counterstain) and anti-GFAP (green, astrocyte marker). Merged images show colocalization. (Down panel) Quantitative analysis of GFAP mean fluorescence intensity (MFI) in olfactory bulb sections by ImagJ. (n =3 mice per group). (****P < 0.0001, ***P < 0.001, **P < 0.01).

**Figure 6:**
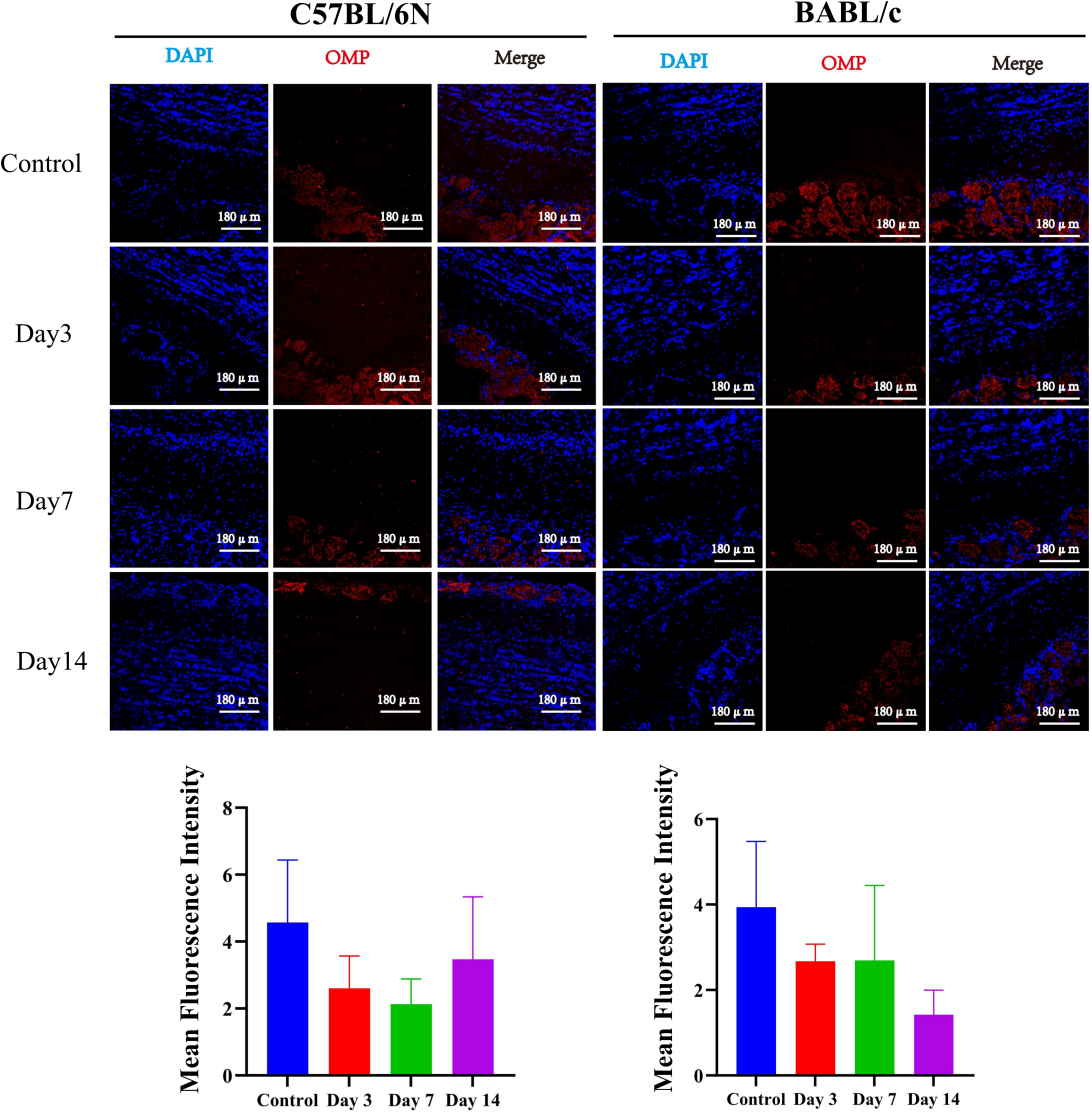
Olfactory nerve injury in the olfactory bulb of C57BL/6N and BALB/c mice following VACV VR-1354 infection assessed by olfactory marker protein (OMP) immunostaining. **(A)** Representative immunofluorescence images of olfactory bulb sections stained with DAPI (blue, nuclear counterstain) and anti-OMP (red, mature olfactory sensory neuron marker). Merged images show colocalization. **(B)** Quantitative analysis of OMP mean fluorescence intensity (MFI) in olfactory bulb sections by ImagJ. (n =3 mice per group).

Astrocyte activation was subsequently examined in the olfactory bulb of C57BL/6N and BALB/c mice following VACV VR-1354 infection. At 3 days post-infection (dpi), astrocytes showed mild activation in both strains. This activation became markedly pronounced at 7 dpi, with elevated levels persisted up to 14 dpi (**Figure 5**). Immunofluorescence staining for olfactory marker protein (OMP), a marker of mature olfactory sensory neurons, revealed functional impairment of mature olfactory sensory neurons in both mouse strains as early as 3 dpi. In C57BL/6N mice, the most severe impairment of mature olfactory nerves was observed at 7 dpi, followed by gradual recovery by 14 dpi. In contrast, BALB/c mice exhibited slightly more pronounced functional impairment at 14 dpi compared to 7 dpi (**Figure 6**).

Collectively, these findings demonstrate that VACV VR-1354 infection in both C57BL/6N and BALB/c mice induces robust activation and proliferation of microglia and activation of astrocytes in the olfactory bulb, accompanied by functional impairment of mature olfactory sensory neurons.

### 3.4 Transcriptome sequencing and analysis of the olfactory bulb from C57BL/6N and BALB/c mice

**Figure 7:**
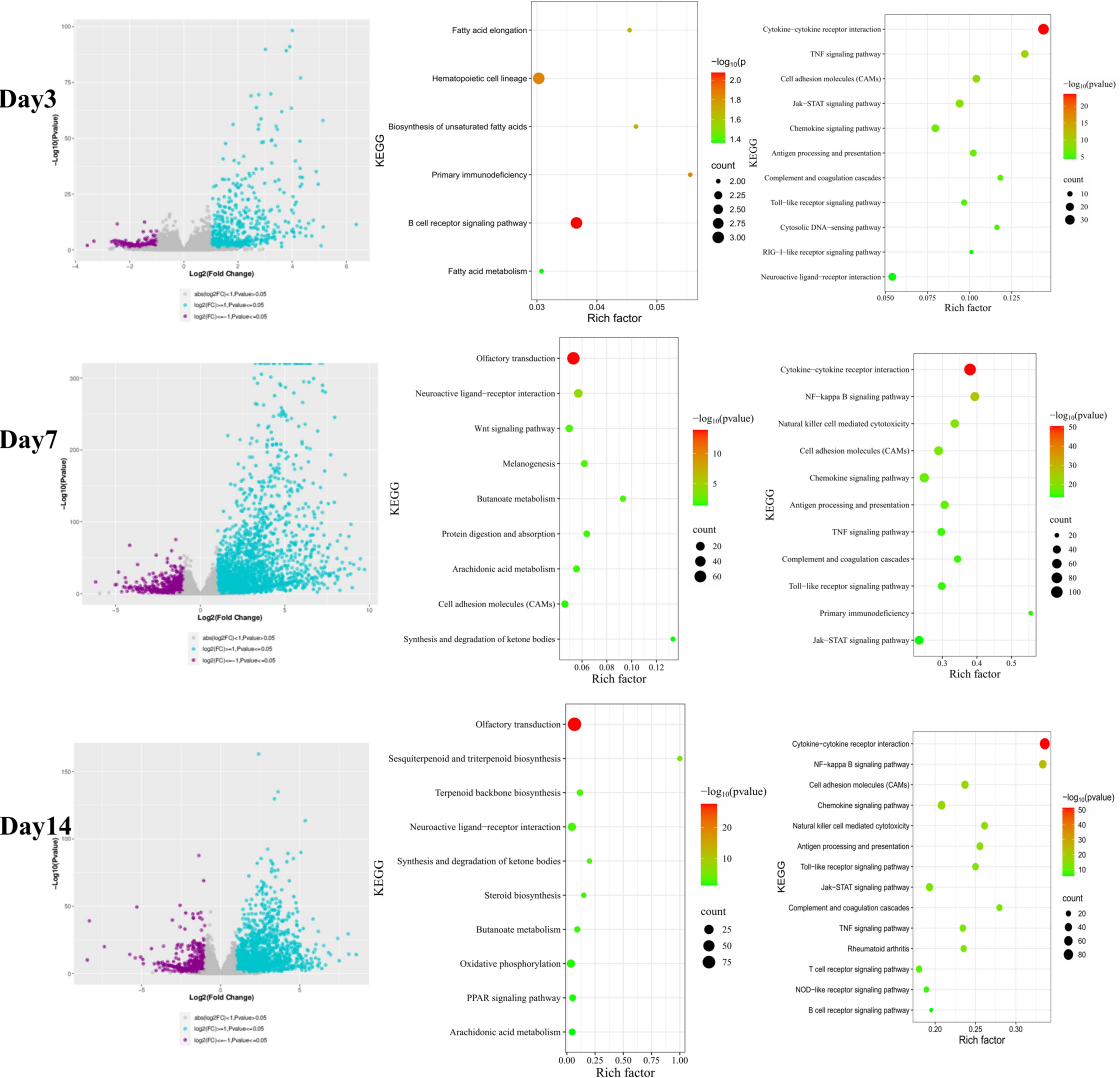
Transcriptome profiling of olfactory bulb tissues from C57BL/6N mice infected with VACV VR-1354. RNA-sequencing was performed on olfactory bulb tissues from infected (n = 5 per time point) and uninfected control (n = 5) C57BL/6N mice at 3, 7, and 14 days post-infection (dpi). (Left panel) Volcano plots depicting differentially expressed genes (DEGs) was conducted using DESeq2 (|log_2_FoldChange| ≥1, and p <0.05). KEGG pathway enrichment was performed on downregulated (Middle panel) and upregulated (Right panel) gene sets.

Transcriptome sequencing was performed on olfactory bulb tissues from C57BL/6N and BALB/c mice at 3, 7, and 14 days post-infection (dpi) with VACV VR-1354. In C57BL/6N mice, RNA-seq analysis of olfactory bulb tissue at 7 dpi identified 1,079 significantly downregulated genes and 3,059 significantly upregulated genes. KEGG pathway enrichment analysis revealed that the downregulated genes were predominantly enriched in the olfactory transduction pathway, including 71 downregulated olfactory receptor (OR) genes (e.g., Or2av9, Or5v1b, Or4z4). In contrast, the upregulated genes were primarily associated with the cytokine–cytokine receptor interaction pathway. At 14 dpi, a total of 873 downregulated and 2,177 upregulated genes were detected. Downregulated genes remained significantly enriched in the olfactory transduction pathway, with 78 OR genes downregulated (e.g., Or4e5, Or52n3, Or10am5), while upregulated genes were again enriched in the cytokine–cytokine receptor interaction pathway (**Figure 7**). At 3 dpi, 282 downregulated and 821 upregulated genes were identified. KEGG analysis showed that downregulated genes were enriched in pathways including hematopoietic cell lineage, whereas upregulated genes were mainly enriched in cytokine–cytokine receptor interaction (**Figure 7**).

**Figure 8:**
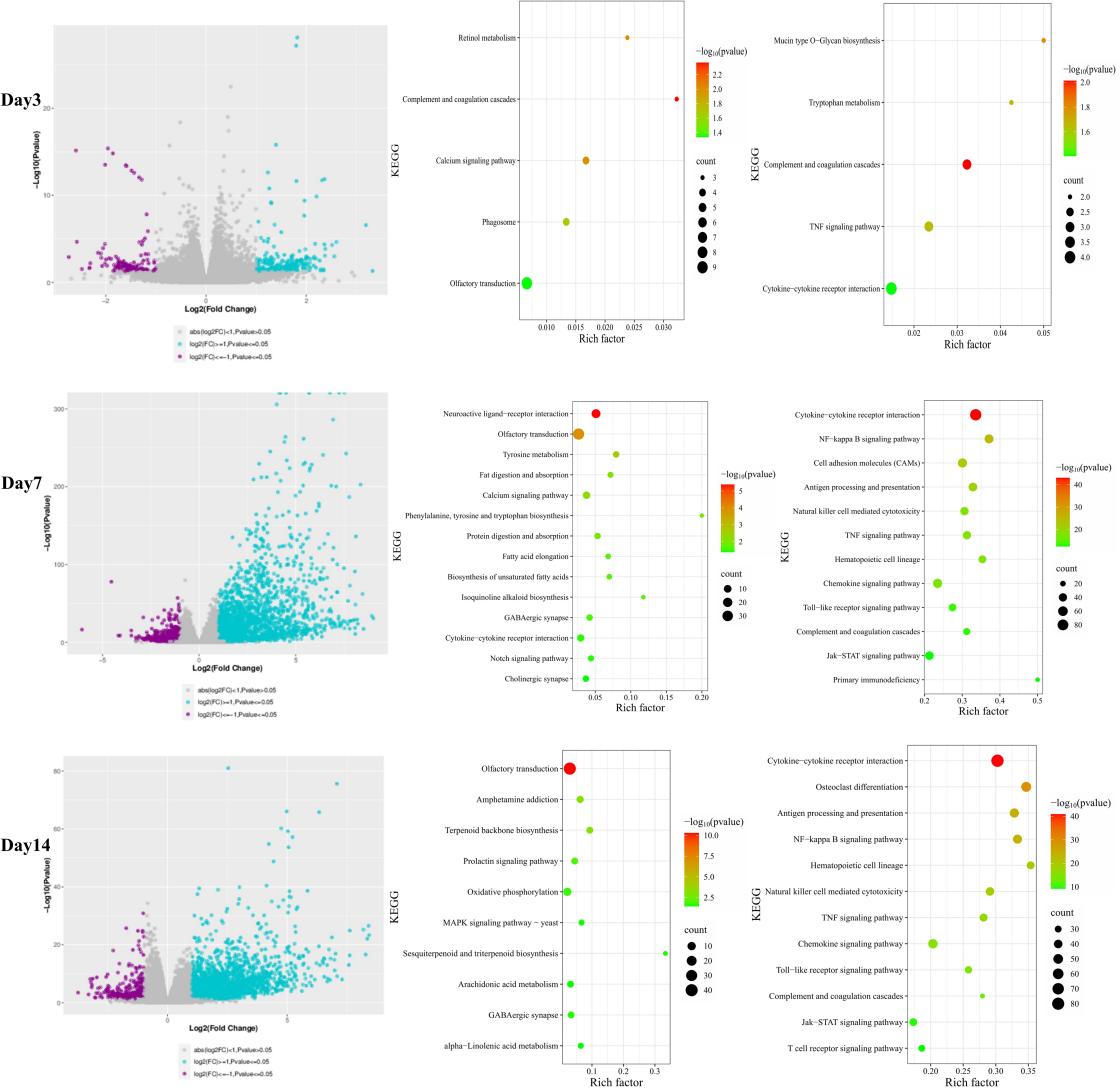
Transcriptome profiling of olfactory bulb tissues from BALB/c mice infected with VACV VR-1354. RNA-sequencing was performed on olfactory bulb tissues from infected (n = 5 per time point) and uninfected control (n = 5) BALB/c mice at 3, 7, and 14 days post-infection (dpi). (Left panel) Volcano plots depicting differentially expressed genes (DEGs) was conducted using DESeq2 (|log_2_FoldChange| ≥1, and p < 0.05). KEGG pathway enrichment was performed on downregulated (Middle panel) and upregulated (Right panel) gene sets.

In BALB/c mice, transcriptomic analysis at 7 dpi revealed 833 downregulated and 2,628 upregulated genes. Downregulated genes were significantly enriched in the olfactory transduction pathway, with 35 OR genes downregulated (e.g., Or4b1, Or8s8, Or12j5). Upregulated genes were predominantly enriched in the cytokine–cytokine receptor interaction pathway. At 14 dpi, 452 genes were downregulated and 2,257 were upregulated. Downregulated genes were again enriched in olfactory transduction, involving 39 downregulated OR genes (e.g., Or2l13b, Or8i2, Or2p2), while upregulated genes were enriched in cytokine–cytokine receptor interaction (**Figure 8**). At 3 dpi, 159 downregulated and 261 upregulated genes were detected. KEGG analysis indicated that downregulated genes were enriched in olfactory transduction and related pathways, with 9 OR genes downregulated (e.g., Or4c103, Or4c112, Or5d18). Upregulated genes were primarily enriched in the cytokine–cytokine receptor interaction pathway (**Figure 8**).

**Figure 9:**
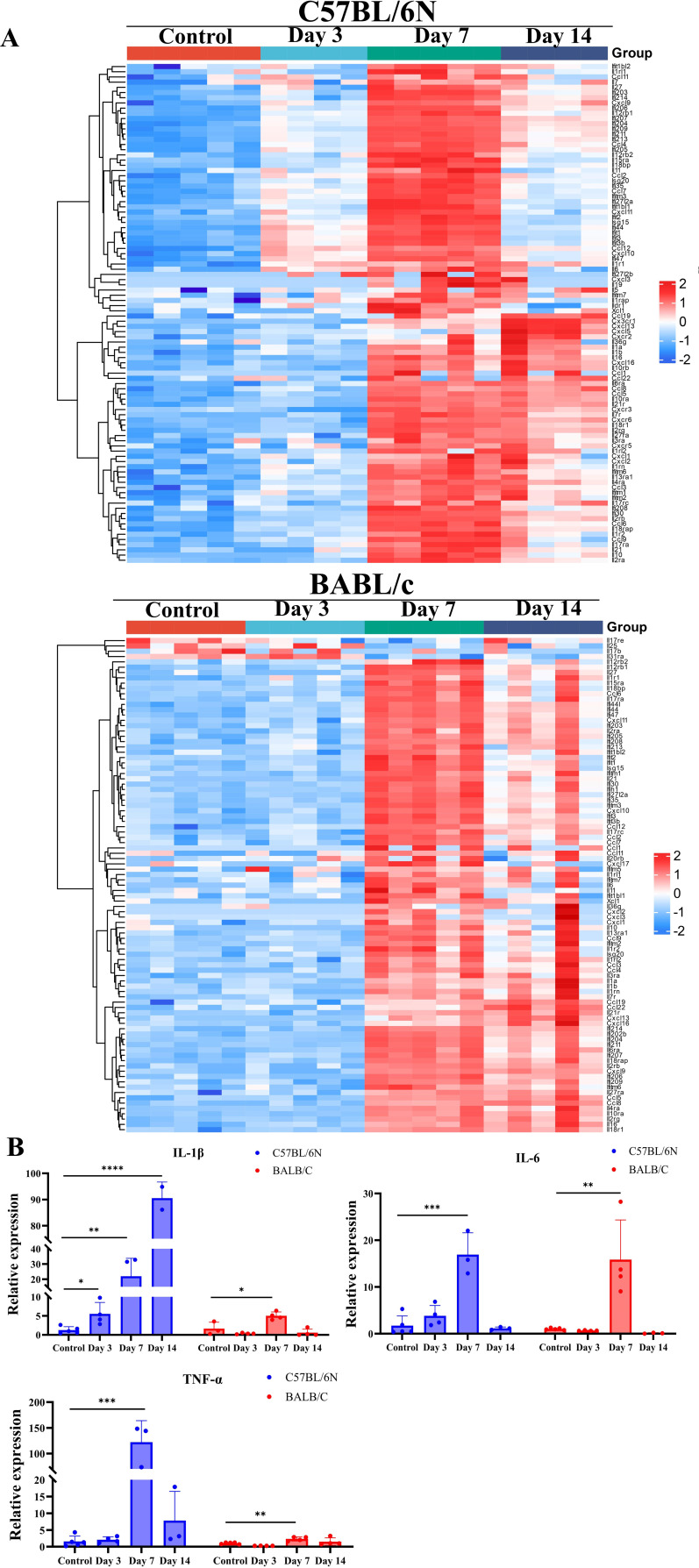
Transcriptome profiling of olfactory bulb tissues from C57BL/6N and BALB/c mice infected with VACV VR-1354. **(A)** Heatmap showing the expression levels of key inflammatory genes, chemokines, and interferon - stimulated genes across 3, 7, and 14 dpi. **(B)** Validation of selected gene expression by Q - PCR (n = 3 per group), confirming the RNA-seq results. (*P < 0.05, **P < 0.01, ***P < 0.001, ****P < 0.0001).

Transcriptomic profiling of olfactory bulb tissues following intranasal VACV-VR1354 infection in both C57BL/6N and BALB/c mice revealed dynamic changes in inflammatory gene expression over different post-infection time points (**Figure 9A**). Specifically, at 3 days post-infection (dpi), nearly no significant changes in inflammatory mediators, chemokines, or interferon-stimulated genes (ISGs) were observed. In stark contrast, by 7 dpi, both mouse strains exhibited significant upregulation of proinflammatory cytokines (log_2_FC ≥ 1 and p < 0.05; e.g., IL-1β, IL-6, TNF-α, IL-1α, IL-10, etc.), chemokines and their receptors (log_2_FC ≥ 1 and p < 0.05; e.g., Ccl2, Ccl5, Cxcl10, Ccr2, Ccr5, etc.), and interferon-stimulated genes (ISGs) (log_2_FC ≥ 1 and p < 0.05; e.g., ISG15, ISG20, Ifi205, Ifi35, Ifi30, etc.). By 14 dpi, some inflammatory genes remained significantly upregulated (e.g., IL-1β, IL-21, IL-27 etc.), while others returned to baseline expression levels, showing no significant difference (e.g., IL-6, IL-11 etc.).

To validate these transcriptomic findings, we performed quantitative real-time PCR (qRT-PCR) on key proinflammatory cytokines (IL-1β, IL−6, TNF−α) across the three time points. Consistent with the RNA-seq results, qRT-PCR confirmed the gene expression trends (**Figure 9B**). These results indicate that the expression trends were consistent with those obtained from transcriptome sequencing.

These results indicate that infection with VACV VR-1354 in C57BL/6N and BALB/c mice leads to downregulation of olfactory receptor genes in the olfactory bulb, and the downregulated genes are related to the olfactory transduction process. Furthermore, the infection triggered a strong immune response in the olfactory bulb, including inflammation and activation of the interferon-mediated antiviral defense mechanism.

### 3.5 Olfactory behavioral analysis in C57BL/6N mice following VACV VR-1354 infection

**Figure 10:**
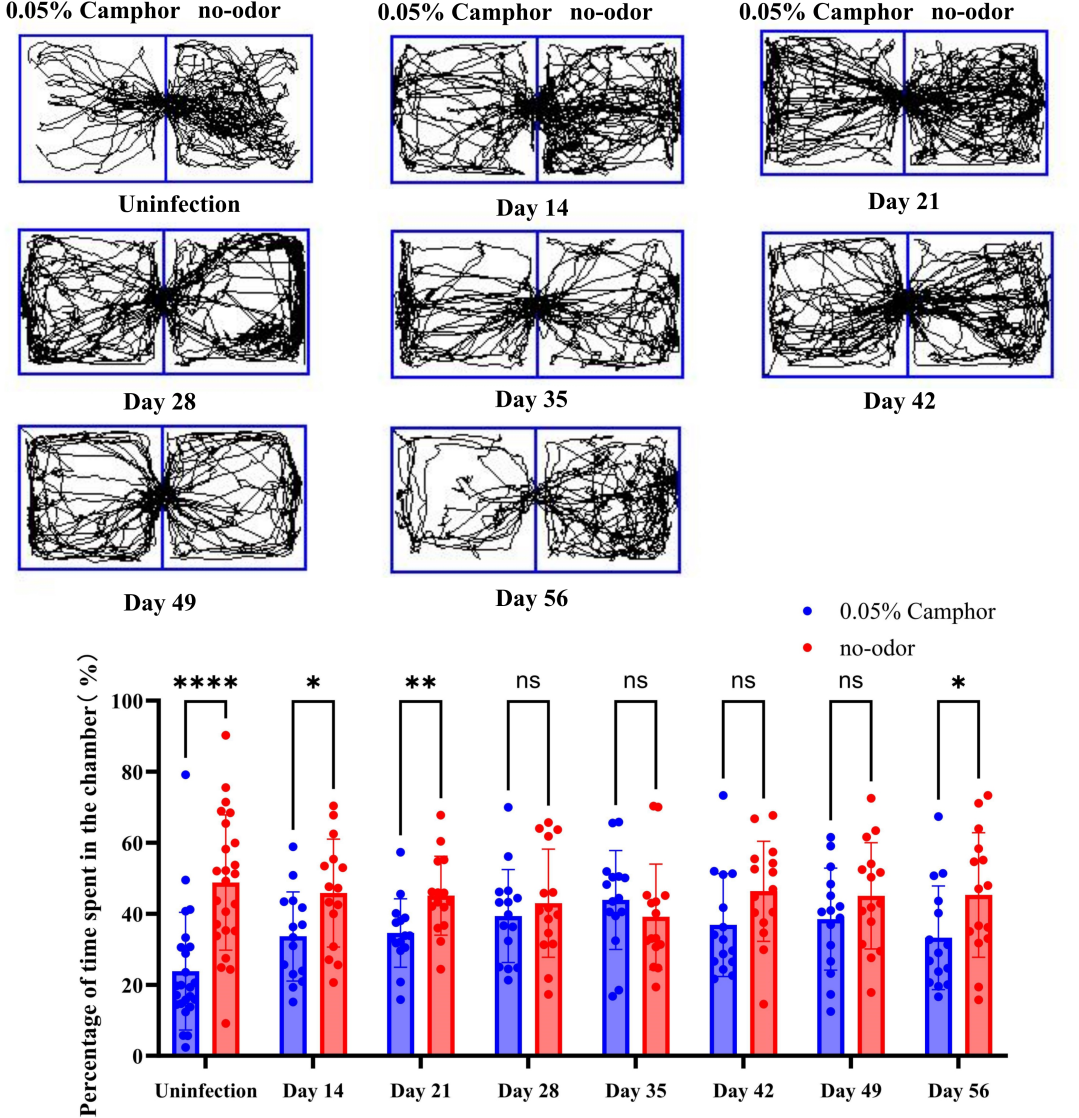
Longitudinal quantification of odor preference deficits in C57BL/6N mice following intranasal VACV VR-1354 infection. (Up panel) Representative tracking paths of mice in a two-compartment chamber: left compartment contained filter paper impregnated with 0.05% camphor solution, right compartment contained odor-free filter paper. (Down panel) Quantitative analysis of time spent in the odor-free compartment, expressed as percentage of total test time. Each data point represents an individual mouse (n = 24 per group); bars indicate mean ± SEM. (*P < 0.05, **P < 0.01, ****P < 0.0001; ns, not significant).

Based on our preliminary data from Evans blue assays and olfactory bulb viral load measurements, which indicated that C57BL/6N mice exhibited more pronounced olfactory phenotype compared to BALB/c mice. we first initiated olfactory behavioral experiments in C57BL/6N mice to establish and optimize the experimental conditions. Olfactory behavioral experiments revealed that uninfected C57BL/6N mice showed aversion to a 0.05% camphor odor. Specifically, the time that the mice spent in the odor - free side of the chamber was significantly longer than that in the side containing 0.05% camphor. At 14 and 21 days post-infection (dpi), the time the mice spent in the odor - free side of the chamber gradually decreased. From 28 to 49 dpi, there was no significant difference in the time the mice spent in the odor - free side and the side with 0.05% camphor. At 56 dpi, the mice gradually resumed their preference for staying mainly in the odor-free side of the chamber. These results suggest that infection of C57BL/6N mice with VACV VR-1354 may impair chemosensory responsiveness to camphor, and this impairment gradually recovers by approximately 56 days post-infection (**Figure 10**).

## 4 Discussion

Central nervous system (CNS) pathology induced by vaccinia virus (VACV), particularly neuroinflammation and functional neurological deficits, remains incompletely understood despite decades of research into this orthopoxvirus. Prior studies have established diverse routes of VACV neuroinvasion, though strain-specific differences in tropism and pathogenesis complicate comparisons. For example, Garcel et al. (18) demonstrated that intravenous inoculation of VACV-WR strain into C57BL/6 mice enables viral migration across the blood-brain barrier (BBB) from the systemic circulation into the CNS. Israely et al. (26) observed robust viral replication and BBB disruption in BALB/c mice following intracranial VACV-WR challenge. In contrast, Hou et al. (15) detected VACV only in the spleen and blood of CAST/EiJ mice after intraperitoneal inoculation, with no evidence of viral neuroinvasion or BBB compromise.

In the present study, we employed an intranasal inoculation model with VACV VR-1354, a tissue-adapted derivative of VACV-WR, to infect two widely used inbred mouse strains—C57BL/6N and BALB/c. We found transient, increased BBB permeability in the olfactory bulb (OB) of both strains, with maximal damage at 7 days post-infection (dpi) and partial resolution by 14 dpi. Notably, BBB permeability was significantly higher in C57BL/6N mice than in BALB/c mice at corresponding time points. Viral load analyses further suggested that high viral titers in both nasal mucosa and OB, with a decreasing gradient of viral distribution (nasal mucosa > OB > cerebrum > cerebellum). Strikingly, C57BL/6N mice exhibited significantly higher viral loads in nasal mucosa and OB compared to BALB/c mice at peak infection, correlating with greater weight loss, mortality, and BBB disruption.

Immunofluorescence analysis revealed synchronized activation of microglia and astrocytes in the OB, initiating at 3 dpi and intensifying at 7 dpi. Microglia exhibited characteristic pro-inflammatory morphological changes, including somal enlargement and retraction of processes, while marked upregulation of glial fibrillary acidic protein (GFAP) in astrocytes confirmed reactive gliosis—a conserved CNS response to injury or infection (27). These neuroinflammatory changes closely paralleled the temporal dynamics of increased BBB permeability, suggesting a causal relationship between viral replication, glial activation, and tissue pathology.

Transcriptomic profiling of the OB provided molecular insights into VACV VR-1354 induced pathogenesis. At 7 dpi, we observed widespread dysregulation of gene expression, with a notable enrichment of downregulated genes in the olfactory transduction pathway—including multiple olfactory receptor (OR) genes. This downregulation persisted at 14 dpi. Given that mature olfactory sensory neurons (OSNs) exclusively express one OR allele each (28), reduced OR transcript levels in the OB likely reflect either impaired anterograde transport from OSN cell bodies in the olfactory epithelium (OE) or transcriptional silencing of OR genes in injured OSNs. Concurrently, upregulated genes were heavily enriched in immune activation pathways, particularly cytokine-cytokine receptor interactions. Quantitative PCR validation confirmed robust induction of pro-inflammatory cytokines (Il1b, Il6, Tnf-α), chemokines (Ccl2, Ccl5, Cxcl10), and interferon-stimulated genes (ISGs), with peak expression at 7 dpi followed by gradual decline. The potent type I interferon response suggested by ISG upregulation likely plays a critical role in limiting viral spread, though excessive pro-inflammatory cytokine production may contribute to neuronal damage and functional deficits.

Behavioral assessment of innate olfactory function using a camphor aversion assay revealed impair chemosensory responsiveness to camphor in infected C57BL/6N mice, beginning at 14 dpi and persisting until 49 dpi, with partial recovery evident by 56 dpi. This delayed recovery timeline contrasts with earlier resolution of viral load and neuroinflammation (by 14 dpi), indicating that chemosensory responsiveness to camphor may require prolonged reinnervation and synaptic remodeling in the OB. Future studies will extend these behavioral assays to BALB/c mice and expand odorant testing beyond camphor to fully characterize strain-specific olfactory deficits. Additionally, single-nucleus RNA sequencing will be employed to dissect cell-type-specific viral tropism and transcriptional responses in the OB, including direct assessment of OSN injury and OR gene regulation.

Taken together, our data identify VACV VR-1354 as a neurotropic poxvirus that efficiently invades the central nervous system via the olfactory route. This invasion triggers transient deficits in chemosensory responsiveness and robust, regionally restricted neuroinflammation—predominantly localized to the olfactory bulb. The tightly coordinated temporal dynamics among viral load kinetics, glial activation, pro-inflammatory gene expression, and behavioral olfactory impairment support a causal model in which acute OB inflammation directly impairs chemosensory function. Functional recovery progresses gradually over approximately 8 weeks.

These findings have broader implications for understanding post-viral chemosensory deficits, a condition with limited therapeutic options. The VACV VR-1354 mouse model recapitulates key features of human post-viral olfactory complaints, including OB involvement, and neuroinflammatory responses. As such, it represents a valuable tool for investigating mechanisms of chemosensory attenuation and to evaluate candidate therapeutics targeting neuroinflammatory resolution. Future work will focus on cell-type-specific viral tropism and transcriptional reprogramming in the olfactory bulb—including direct assessment of olfactory sensory neuron (OSN) injury, dysregulation of odorant receptor gene expression, and perturbations in camphor-responsive signal transduction pathways. Concurrently, we will test strategies to promote olfactory sensory neuron regeneration during camphor-responsive functional recovery.

## Data Availability

The datasets presented in this study can be found in online repositories. The names of the repository/repositories and accession number(s) can be found below: https://db.cngb.org/cnsa/, CNP0008526.
